# Comparison of ELISA with automated ECLIA for IL-6 determination in COVID-19 patients: An Italian real-life experience

**DOI:** 10.1016/j.plabm.2024.e00392

**Published:** 2024-04-01

**Authors:** Francesca Romano, Luisa Lanzilao, Edda Russo, Maria Infantino, Francesca Nencini, Giovanni Cappelli, Stefano Dugheri, Mariangela Manfredi, Alessandra Fanelli, Amedeo Amedei, Nicola Mucci

**Affiliations:** a General Laboratory, Azienda Ospedaliero-Universitaria Careggi, Florence, Italy; b Department of Experimental and Clinical Medicine, University of Florence, 50121, Florence, Italy; c Immunology and Allergology Laboratory Unit, S. Giovanni di Dio Hospital, 50143, Florence, Italy

**Keywords:** COVID-19, Interleukin-6, ELISA, ECLIA

## Abstract

**Objectives:**

Coronavirus disease 2019 (COVID-19) has a wide spectrum of clinical severity. A cytokine storm is associated with COVID-19 severity. Of these, IL-6 is significantly associated with higher mortality and is also a marker for predicting disease prognosis. IL-6 may act as a target for therapeutics and, a blockade of IL-6 function by Tocilizumab has been described as a treatment of the inflammatory process COVID-19-related. This study aims to describe our experience comparing two different methods, in detail Human IL-6 Instant ELISA and the Elecsys IL-6 based on ECLIA, for the IL-6 assessment.

**Design and methods:**

IL-6 levels from serum samples of 104 COVID-19 patients, admitted to the AOU Careggi (Hospital in Florence -Italy), were assessed by using the two above-mentioned methods, and the results were analysed through Passing-Bablok regression fit and Bland-Altman plot.

**Results:**

The regression exhibited a linear relation between the methods with a regression equation (y = - 0.13 + 0.63 x; 95 % C.I. intercept = − 0.13 to 4.55; 95 % C.I. slope = 1.03 to 1.26 with R^2^ = 0.89, p > 0.05), showing a positive slope. The agreement of the two methods reported a bias of −25.0 pg/mL. Thus, the two methods correlate but do not agree in terms of numeric results.

**Conclusions:**

The two assays showed good comparability. However, because of the extremely wide linear range of the ECLIA, its throughput and its capacity for immune profiling, it represents an interesting emerging technology in the immunology field.

## Highlights

• A cytokine storm is associated with COVID-19 severity.
• IL-6 is associated with higher mortality and it is a robust prognostic marker.
• Human IL-6 Instant ELISA Kit (Invitrogen) and the ECLIA Elecsys IL-6 (Roche Diagnostics) were tested for the IL-6 assessment.
• A linear relation between ELISA and ECLIA was observed.
• The ECLIA method represents an interesting technology in the immunology sector.

## 1 Introduction

Severe acute respiratory syndrome coronavirus-2 is the causative pathogenic agent of the coronavirus disease 2019 (COVID-19), which was declared a pandemic by the World Health Organization (WHO) in March 2020 and still is a major concern worldwide. From the very beginning, broadly disseminated immune dysregulation, characterized by the cytokine storm, was described as an important component of the pathophysiology of COVID-19, and the interleukin- 1 (IL-1) –interleukin-6 (IL-6) axis was described as one of the most biologically relevant signalling pathways in the SARS-CoV-2-induced hyperinflammatory reaction, finding a high correlation between IL-6 in serum and COVID-19 prognosis [[1], [2], [3], [4]].

Indeed, many studies have demonstrated that IL-6 is significantly elevated in patients with COVID-19 and is predictive of poor outcomes [5]. These features provided the rationale for the immunotherapeutic approach, taken into consideration to counter the hyperinflammation in severe COVID-19. So, during the pandemic, IL-6 “exploded” among COVID-19 markers, although it was already largely used as a general inflammatory marker in patients with immune and rheumatic diseases [[6], [7], [8], [9]]. In particular, its application is related to the therapy with Tocilizumab (TCZ) [1], an antagonist of the IL-6 receptor (IL-6R), and with the commonly used corticosteroids [10]. Regarding the experience of the Careggi University Hospital (Florence, Italy), before the COVID-19 pandemic, IL-6 detection was performed in the laboratory of Immunology through an Enzyme-Linked immunosorbent assay (ELISA), only for immune disease conditions, with an average of 4 h for the execution and waiting times up to 14 days. However, during the pandemic, the requests for IL-6 detection increased exponentially, but the ELISA method was no longer the method suitable for the urgent context of COVID-19. For this reason, an automated electrochemiluminescence immunoassay was implemented as an automated test h24, to monitor the effect of TCZ in COVID-19 patients. Indeed, TCZ has shown beneficial effects in reducing the severity and mortality of severe COVID-19 [11,12]. In this context, serial measurements of IL-6 in the serum or plasma of patients were shown to be useful in evaluating the severity of the disease and predicting the outcome of these patients, helping to guide a personalized therapy, providing prognostic information, evaluating response to therapy, predicting complications as well as developing organ dysfunction.

Therefore, given the strong reduction of the laboratory turnaround time (TAT) (about 18 min to have a result) and as a consequence, of the severity and mortality of COVID-19, the Electro-Chemiluminescence Immunoassay (ECLIA) was used in all the hospitalized and emergency room COVID patients [13].

Given the little literature data on the comparison between the ELISA and ECLIA methods, we aimed to describe our real-life experience with the evaluation of the harmonization between the ELISA and ECLIA methods during the first wave of COVID-19 pandemic in Italy.

## 2 Materials and methods

### 2.1 Patient recruitment

We conducted a retrospective cohort study on 104 COVID-19 patients who were consequently admitted to the Emergency and Medicine Department of the University Careggi Hospital (Florence, Italy) between April and May 2020.

COVID-19 diagnosis was assessed according to the CDC guidelines updated in April 2020 by Nucleic Acid Amplification Testing (https://www.cdc.gov/coronavirus/2019-ncov/lab/resources/antigen-tests-guidelines.html).

This study was approved by the Institutional Review Board Committee (17 104_oss), and informed consent was obtained from all participants. The comparison study was evaluated by using serum samples to assess IL-6 concentration.

### 2.2 Analysis of serum samples for IL-6 concentrations

Serum samples were assessed in parallel using both the automated ECLIA Elecsys® IL-6 – (Roche Diagnostics, Basel, Switzerland) on the immuno-analyzer Cobas 8000 platform (Roche Diagnostics, Basel, Switzerland) and the Human Instant ELISA™ (Invitrogen, Waltham, Massachusetts, United States). Both methods use monoclonal antibodies raised against epitopes of recombinant IL-6.

### 2.3 Elecsys® IL-6

The Elecsys® IL-6 assay is a non-competitive (sandwich) chemiluminescent immunoassay. 18 μL of sample undergoes a first incubation with IL-6 specific antibodies, followed by a second incubation with IL-6 specific antibodies labelled with ruthenium complexes to form a sandwich complex. Thereafter, complexes are magnetically captured, where a voltage then induces a chemiluminescent emission directly proportional to the IL-6 concentration. Results are determined via a calibration curve which is instrument-specifically generated by 2-point calibration and a master curve provided via the reagent barcode or e-barcode. The assay has a claimed measuring range of 1.5–5000 pg/mL, which could be extended to 50 000 pg/mL with a 10-fold dilution of the sample; the coefficients of variation for inter-assay and intra-assay precisions were <10% and the analytical sensitivity of the test was 1.5 pg/mL.

### 2.4 Human Instant ELISA™

Human Instant ELISA™ was supplied by Invitrogen. An anti-human IL-6 coating antibody is adsorbed onto microwells. Human IL-6 present in the sample or standard binds to antibodies adsorbed to the microwells; a biotin-conjugated anti-human IL-6 antibody binds to human IL-6 captured by the first antibody. Streptavidin-HRP binds to the biotin conjugated anti-human IL-6. The first incubation lasts 3 h on a microplate shaker at room temperature (18 °C–25 °C). A colored product is formed in proportion to the amount of soluble human IL-6 present in the sample. The reaction is terminated by the addition of an acid and absorbance is measured at 450 nm. A standard curve is prepared from 7 human IL-6 standard dilutions to determine human IL-6 sample concentration determined. Seven standards containing 3.1,6.3, 12.5, 25.0, 50.0, 100.0 and 200.0 pg/ml of recombinant IL-6 were used for calibration. Each sample, standard, blank and control sample should be assayed in duplicate. The immunoassay is calibrated with highly purified recombinant human IL-6 which has been evaluated against the international Reference Standard NIBSC 89/548 and has been shown to be equivalent. NIBSC 89/548 is quantitated in International Units (IU), 1IU corresponding to 10 pg human IL-6. The basic measuring range was 3.1–200 pg/mL; the coefficients of variation for interassay 7% and intra-assay precisions were 6.2 %. The analytical sensitivity of the test was 0.92 pg/mL.

### 2.5 Statistical analyses

The agreement between Elecsys® IL-6 vs Human Instant ELISA™ was analysed through Passing-Bablok regression fit and Bland-Altman plot. In particular, we first performed the Passing-Bablok regression fit using the website application https://bahar.shinyapps.io/method_compare/{Passing, 1983 #6027}. Passing-Bablok regression comparison was considered statistically significant when p value < 0.05.

In addition, the Bland-Altman plot described the agreement between the two quantitative measurements by using the mean and the differences [[14], [15]] and methods concordance was considered statistically significant when a p value p > 0.95 was obtained by comparing the mean of the differences vs.0.

## 3 Results

To compare the two methods, we assessed relationship and agreement by using the Passing- Bablok regression and the Bland – Altman in 104 COVID-19 patients’ serum samples.

**Fig. 1:**
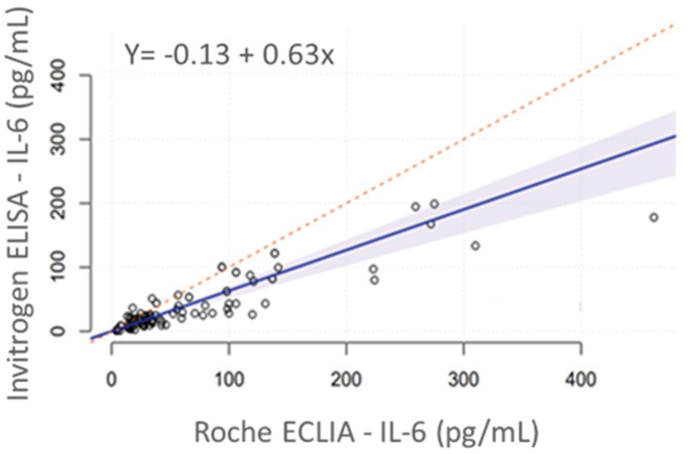
Passing-Bablok regression fit of IL-6 concentrations obtained by Human Instant ELISA™ and Elecsys® IL-6 over the 104 samples. The dashed line represents the optimal regression line. The solid line represents the best fit by linear regression. R2 is the Pearson linear regression coefficient. Y is the constant bias and x is the slope of the regression line.

The Passing - Bablok regression exhibited a linear relation between ELISA and ECLIA with a regression equation (y = - 0.13 + 0.63 x; 95 % C.I. intercept = − 0.13 to 4.55; 95 % C.I. slope = 1.03 to 1.26 with R^2^ = 0.89, p > 0.05), showing a positive slope (Fig. 1).

However, the IL-6 median of all patients (total n = 104) was 22.4 pg/mL and 32.3 pg/mL for ELISA and ECLIA, respectively. In addition, the first (9.8 pg/mL for ELISA versus 18.75 pg/ml for ECLIA) and third quartile (43.42 pg/mL for ELISA versus 87.9 pg/mL for ECLIA), reported a constant ECLIA higher level of IL-6 concentration, corresponding to the approximately 14.4% for all values obtained.

**Fig. 2:**
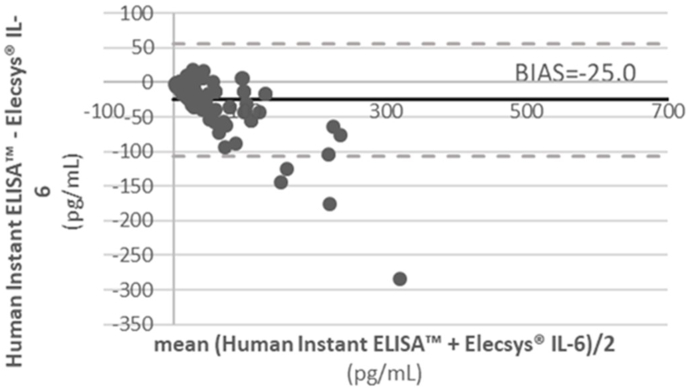
Bland-Altman plot of the different values obtained by Human Instant ELISA™ and Elecsys® IL-6. The black continuous line represents the bias (mean difference), and the grey lines are the limits of agreement (mean ± 2SD) over the 104 samples.

Bland-Altman analysis showed the mean difference (Bias) ± upper and lower limits (Fig. 2). Precisely, the agreement of the two methods reported a bias of −25.0 pg/mL.

**Fig. 3:**
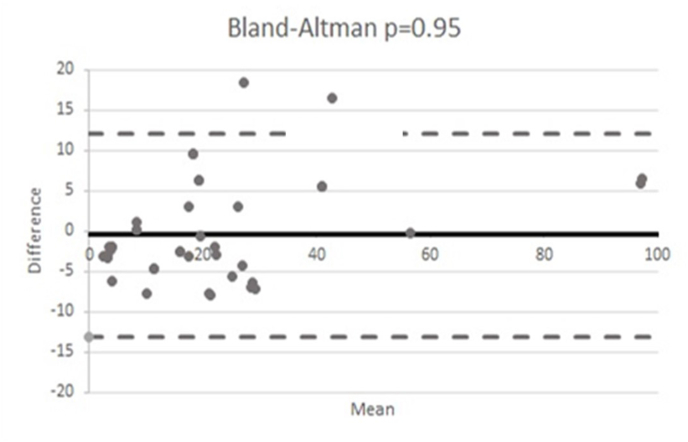
Bland-Altman plot for samples with agreement of the results by Human Instant ELISA™ and Elecsys® IL-6. The black continuous line represents the bias (mean difference), and the grey lines are the limits of agreement (mean ± 2SD) over the 42 samples). Each dot represents the difference between samples measured with the two above-mentioned methods.

Further analysis was performed to deepen how many values reached an agreement for both the two methods and, in particular, statistically significant agreement was found for 42 patients, with value in ELISA in the range of 0.8–100.6 pg/mL (mean 22.27 pg/mL) and 3.99–94.0 pg/mL (mean: 22.71 pg/mL) in ECLIA (Fig. 3). In detail, the plot showed us the trend of differences with only 50% of the points within the 95% of confidential interval (95% CI) for all range of concentrations.

## 4 Discussion

In this study, our objective was to document our practical observations regarding the alignment of the ELISA and ECLIA methods in detecting serum IL-6 levels during the initial surge of the COVID-19 pandemic in Italy, conducted at Careggi University Hospital in Florence (Italy).

As previously reported, IL-6 plays a critical role in triggering the cytokine storm observed in COVID-19 patients, and it may serve as a predictive marker for respiratory failure, aiding in the stratification of patients who could potentially benefit from early anti-IL-6 treatment [16]. Before the onset of the COVID-19 pandemic, IL-6 detection was exclusively conducted at the Immunology Laboratory of Careggi University Hospital using ELISA. This method was primarily reserved for immune-related conditions, resulting in prolonged average execution and waiting times. Throughout the pandemic, there was a notable surge in the requirement for IL-6 detection within our hospital. However, the ELISA method was no longer feasible for the emergency. Consequently, we decided to implement the ECLIA test h24, previously used to monitor the effect of TCZ in COVID-19 patients. To assess the comparability between the two methods, in our real-life experience we performed the Passing-Bablok regression fit and Bland-Altman plot during the first wave of the COVID-19 pandemic in Italy, evaluating the harmonization between the two methods.

In general, the IL-6 assay run on the full-automated platform by the ECLIA method has great applications in most laboratories for rapid diagnosis and monitoring of the immune response in COVID-19 patients. This facilitates the selection of appropriate treatments in patients with hyper-inflammation. The complete automation of the ECLIA method, in contrast to ELISA, streamlines and expedites IL-6 testing in emergency laboratories, ensuring 24-hour availability of results. Additionally, the ECLIA method offers a broader range of measurement compared to ELISA, further enhancing its applicability in clinical settings.

## 5 Conclusions

In our experience, the two assays showed good comparability. Given the extremely wide linear range of the ECLIA, and its throughput and capacity for immunoprofiling it represents an emerging technology in the immunology field, but future studies are needed to fine assess the clinical performance of the new fully automated ECLIA methods in large sample cohorts.

## Ethical approval and consent to participate

The Author ensure that all procedures were performed in compliance with relevant laws and institutional guidelines and the study have been approved by Institutional Review Board Committee (17 104_oss), and informed consent was obtained from all participants.

## Funding

This research did not receive any specific grant from funding agencies in the public, commercial, or not-for-profit sectors.

## CRediT authorship contribution statement

**Francesca Romano:** Conceptualization, Data curation, Writing – original draft. **Luisa Lanzilao:** Conceptualization, Data curation, Writing – original draft, Formal analysis. **Edda Russo:** Conceptualization, Data curation, Formal analysis, Writing – original draft, Writing – review & editing. **Maria Infantino:** Data curation, Formal analysis. **Francesca Nencini:** Formal analysis, Visualization. **Giovanni Cappelli:** Visualization, Writing – original draft. **Stefano Dugheri:** Methodology, Software. **Mariangela Manfredi:** Supervision, Writing – review & editing. **Alessandra Fanelli:** Supervision. **Amedeo Amedei:** Supervision. **Nicola Mucci:** Supervision.

## Declaration of competing interest

Francesca Romano: None.

Luisa Lanzilao: None.

Edda Russo: None.

Maria Infantino: None.

Francesca Nencini^:^ None.

Giovanni Cappelli: None.

Stefano Dugheri: None.

Mariangela Manfredi: None.

Alessandra Fanelli: None.

Amedeo Amedei: None.

Nicola Mucci: None.

## Data Availability

No data was used for the research described in the article.
